# Morphometric Examination of the Sacroiliac Region and Variable Positions of the Sacral Auricular Surface: Anatomical Classification and Importance

**DOI:** 10.7759/cureus.33792

**Published:** 2023-01-15

**Authors:** Ömer Faruk Cihan, Mehmet Karabulut

**Affiliations:** 1 Anatomy, Gaziantep University, Gaziantep, TUR; 2 Anatomy, Selçuk University Faculty of Medicine, Konya, TUR

**Keywords:** impression, sacroiliac joint area, auricular surface, sacroiliac joint, sacrum

## Abstract

Objectives: Anatomical variations of the sacrum involve alterations in the number of sacral segments, auricular surface area, and neural arch dimensions and are associated with biomechanical, surgical, and obstetric implications. Due to the complex functional structure of the sacroiliac (SI) joints, it was aimed to evaluate their morphometric structure, classify the sacrum, and group different locations of the auricular surface in the sacrums.

Methods: Sex determination was made in 91 dry human sacra. Determination of the alpha angle on the sagittal axis of the auricular surface of the sacrum and classification of the auricular surface of the sacrum was performed. The sacra were studied to determine the position and extent of their auricular surfaces in relation to the sacral segments. Specimens were grouped as “normal,” “high-up,” and “low-down” auricular surface-bearing sacra. The sacral surface areas were measured, and SI joints were classified into three types morphologically (types I, II, and III). The depth and anterior-posterior length of the cranial, middle, and caudal impressions observed in the posterior sacrum and anterior-posterior lengths were measured.

Results: The measurements made for sex determination showed that 46 of the sacra were from females and 45 were from males. The alpha angle on the sagittal axis of the sacral auricular surface was found to be greatest at the level of the first posterior sacral foramina. While the most common sacrum type was Type III, the least common type was Type I. The surface area of facies auricularis was found to be larger in males than in females. With regard to facies auricularis in all sacrum groups, although it covered approximately 2.5 sacral vertebrae, there was a difference in the location of facies auricularis in the sacral vertebrae. A statistically significant difference was found between right and left in the depth values of the impressions in the dorsal surface of the sacrum (p <0.05).

Conclusion: The position of the auricular surface in the sacrum differed among individuals. These differences are associated with variable load-bearing in the SI joints. The biomechanical classification of the sacrum and localization of the auricular surface can provide information about the anatomic source of low back pain or help predict the location of low back pain. Changes in the synovial surface morphology of the SI joints may elicit sacroiliac joint pain. This study was conducted because it is considered that the location of the auricular surface can significantly affect load-bearing patterns of the sacrum.

## Introduction

The sacroiliac (SI) joints can be regarded as a highly “dynamic” structure in the sense that they undergo a wide range of morphological changes throughout life [1]. In the development stage of the bone, some changes occur in the morphology and properties of the SI joints. Although it is generally agreed that surface irregularities of the SI joints are common anatomical features that occur later in life, both elevations and depressions have been reported in the dorsal side of the sacrum in young individuals. Given the frequency of surface changes reported in the literature, many researchers stated that changes in the joint cartilage are age-related normal changes due to accumulated stress. Among these changes, impressions occur in response to stress and, at the same time, create a functional lock between the bones [2-5]. This locking serves to optimize joint stability by limiting movement in the joint. Thus, dislocations in the joint are greatly reduced due to increased stability of the SI joints. It has been stated that the surface morphology of the SI joints is probably related to the posture of the individual, and therefore, alterations in the surface shape may be related to the spinal curvature [4-7]. Since the SI joints act as vital linking stations of the pelvic bony ring, mobility at the pelvis has been associated with the range of movement at the SI joints, exclusively in the process of parturition [8]. Movement of the SI joint is more complex than simple uniaxial movements [1,9]. For clinicians, the position of the auricular surface may be important in understanding and evaluating the morphology of the sacrum. The surface position of the auricular surface may vary depending on the sacral body of the individual. Differences in the surface positions of the auricular surface may occur due to transition anomalies in lumbosacral junctions. Although the surface area of the auricular surface determines the magnitude of weight transfer to the hip bone, it can also be important in carrying the load on the sacrum [10-12]. Jesse et al. categorized the variability of the morphology of the SI joints into three types according to the alpha angle on the sagittal axis of the joint. They noted that the variability in the synovial surface morphology of the SI joint may predispose to the development of low back pain [13].

Recognition of the normal anatomical structure and morphometry of the sacrum can prevent complications that may occur during surgical procedures in this area. We think that certain anatomical parameters, those related to the S2 vertebra, in particular, may be important for the spinal instrumentation procedure used by spine surgeons. To provide adequate morphological data to assist anatomists and clinicians, the aims of the study were 1) to identify the primary changes in the position of the auricular surface of the sacrum in relation to the sacral vertebrae, 2) to categorize sacrums according to the position of the auricular surface in the sacral vertebrae, 3) to measure the surface area of the auricular surface, and 4) to evaluate the morphology of the sacrum. We carried out this study because we considered that it would provide a different perspective to the studies in the literature by measuring the morphological structures of the sacrum.

## Materials and methods

**Figure 1:**
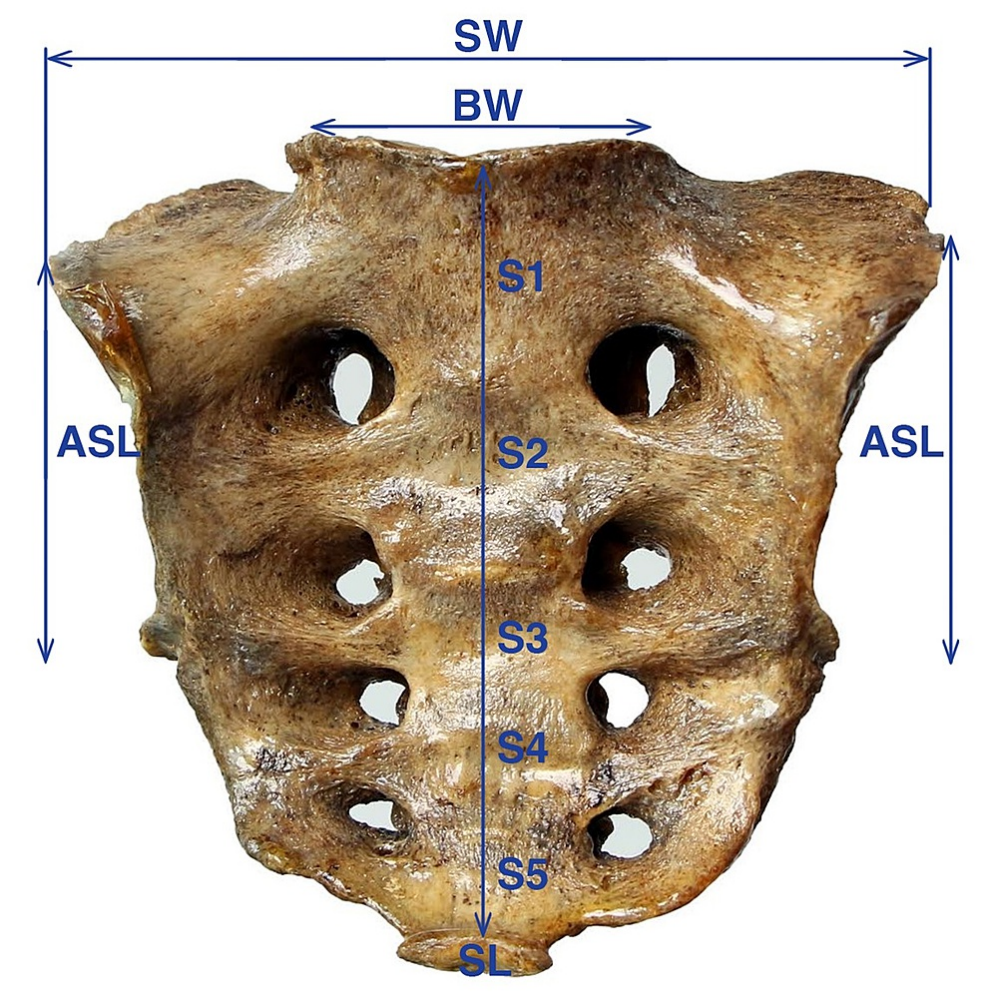
Measurements of sacral parameters for sex determination. SW: sacral width; BW: body width of S1 vertebral body. ASL: auricular surface length; SL: sacral length.

The following measurements were obtained on 91 sacrums with undetermined age, archived in the laboratories of the Departments of Anatomy at Gaziantep University and Çukurova University Faculties of Medicine (Southern Turkey). Length measurements on the bones were obtained using a digital caliper (Mitutoyo, Japan) with an accuracy of 0.01 mm. For the sex determination of the sacrum, the widest distance (sacral width) connecting the points in the upper lateral part of the ala of the sacrum and the distance between the base of the sacrum and the apex of the sacrum (sacral length) were measured with the caliper (Figure 1). Sex was determined with a 99.75% accuracy rate using the following formula reported in the literature: sacral index = sacral width X 100/sacral length. The sacral index was 90.5 to 106 mm in males and 104.8 to 131 mm in females. Sacral index values of <96.03 for males and > 110.05 for females have been determined as definite cut-off values for sex determination [14-16].

Photographs of the sacrums were taken with a Sony DSC-HX100V 30x optical zoom camera (Minato, Japan) from a distance of 30 cm at standardized positions suitable for study, and the camera was fixed. After that, the images were transferred to a computer, and the measurements described below were obtained using the Horos v.3.0.1 software (Annapolis, USA) package. The determination of the alpha angle on the sagittal axis of the auricular surface of the sacrum and the classification of the auricular surface of the sacrum were conducted as described by Jesse et al. [13]. To calculate the alpha angle, two lines were drawn along the axis of the anterior and posterior extensions of the auricular surface of the sacrum.

**Figure 2:**
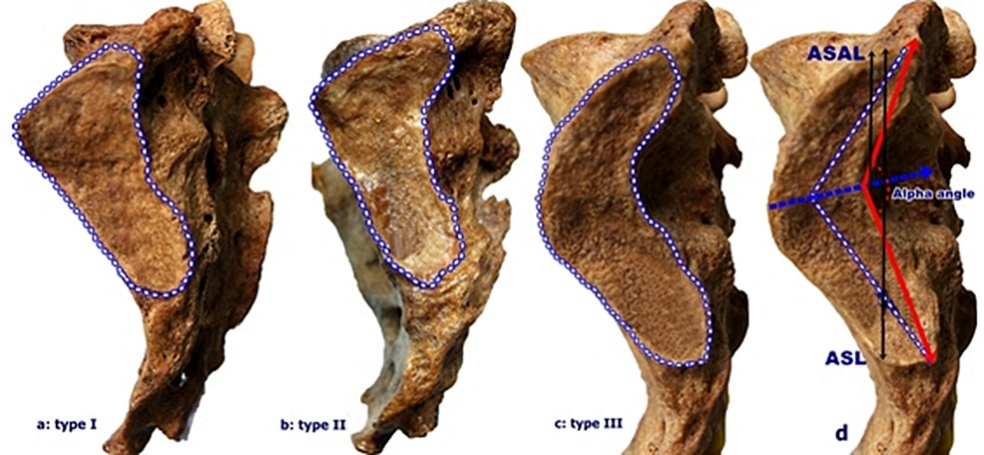
Classification of sacrum according to the alpha angle on the joint surface (a, b, c) Representation of Type I (a-scone-shaped), Type II (b-auricular-shape), and Type III (c-crescent-shaped) sacroiliac joint morphology and d. Determination of the alpha angle of the auricular surfaces, the length of the auricular surfaces (ASL), and the length of the upper border of the auricular surfaces to the alpha angle (ASAL) are shown [13].

Classification of the joint surface of the sacrum was performed according to the alpha angle defined above. The sacrum was classified as type I (scone-shaped) when the alpha angle was> 160º, type II (auricular-shape) when the alpha angle was 130º - 160º, and type III (crescent-shaped) when the alpha angle was <130º (Figures 2a-2c). The alpha angle was formed by two lines that intersect at the maximum nutation point in the posterior part of the auricular surface of the sacrum, starting from the middle of the distal parts of the anterior and posterior appendages.

To determine the auricular surface length (ASL), the distance between the upper and lower limits of the auricular surface was measured. In addition, the length of the upper boundary of the auricular surface to the alpha angle (ASAL) was measured [13].

The digital images were transferred to the computer for surface area measurements of the auricular surface and calibrated using the Horos v.3.0.1 software. After calibration, the surface area value was calculated as cm2 using the relevant feature of Horos v.3.0.1.

**Figure 3:**
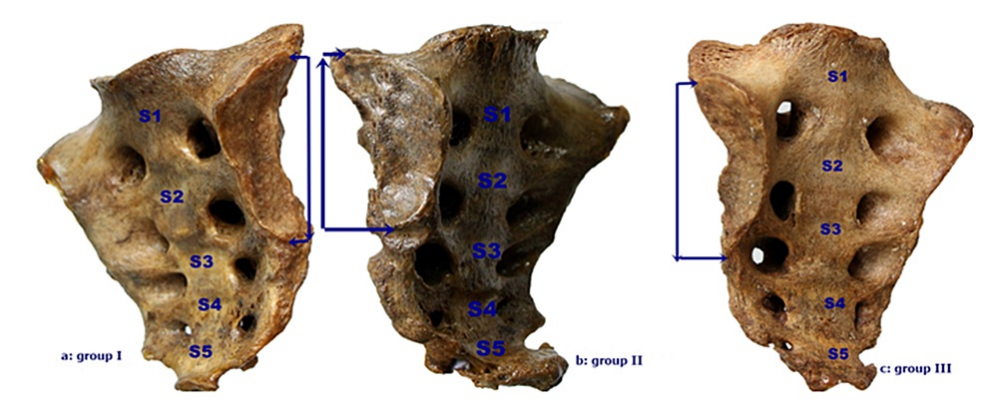
Grouping of sacrum according to the position of the auricular surface in the sacrum.

The sacrum was divided into three groups, as described by Mahato, according to the position of the auricular surface in the sacral vertebrae. Group I (Normal): The location of the auricular surface extends from S1 vertebra level to the middle of the S3 vertebra. Group II (High-up): The location of the auricular surface starts above the S1 vertebra level and extends to the upper levels of the S3 vertebra. Group III (Low-down): The location of the auricular surface starts from the lower part of the S1 vertebra and extends to the lower part of the S3 vertebra (Figures 3a-3c) [10].

**Figure 4:**
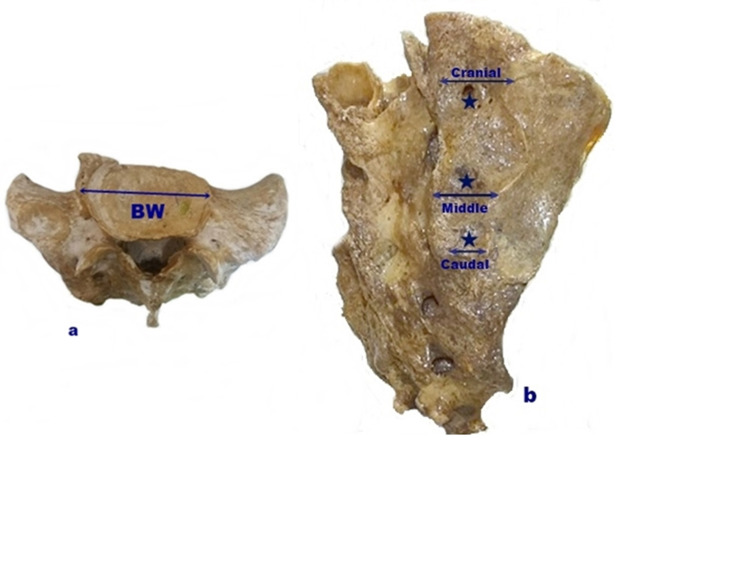
Representation of the body width of the S1 vertebral body and impression a: Body width of the S1 vertebral body (BW), b: Stars show the depths of cranial, middle, and caudal impressions in the dorsal surface of the sacrum, and arrows show the anterior-posterior length of the impressions.

The transverse diameter of the S1 vertebral body was measured as the longest transverse distance of the base of the sacrum at the S1 level. The depth and anterior-posterior length of the cranial, middle, and caudal impressions observed in the posterior of the sacrum were measured (Figure 4).

Statistical analysis

Pearson’s correlation analysis was used to investigate the relationship between two numerical variables. One-way ANOVA was used to compare parameters among more than two groups. Whether the data followed a normal distribution was tested using the Shapiro-Wilk test. Mann-Whitney U test was employed to compare non-normally distributed parameters between two independent samples and the Wilcoxon test was used to compare two dependent samples. Relationships between categorical variables were examined using the chi-squared test. Descriptive statistics were presented as mean ± standard deviation for numerical variables and as numbers and percentages for categorical variables. IBM Corp. Released 2013. IBM SPSS Statistics for Windows, Version 22.0. Armonk, NY: IBM Corp. software package was used for statistical analysis of the study data. A p-value less than 0.05 was considered statistically significant.

## Results

The measurements made for sex determination showed that 46 (50.5%) of the sacrums were from females, and 45 (49.5%) were from males. The alpha angle on the sagittal axis of the sacral auricular surface was found to be greatest at the level of the first posterior sacral foramina. The alpha angle on the right and left joint surfaces were more commonly located at the level of the first sacral dorsal foramina in females than in males at the dorsal level. However, no significant difference was observed between the sexes (right alpha angle p = 0.801, left alpha angle p = 0.254) (Table 1, Figure 2).

**Table 1:** Comparison of the level of alpha angle on the right and left joint surfaces by sex.

Parameters	Male				Female				Total			
	Number (n)		Frequency (%)		Number (n)		Frequency (%)		Number (n)		Frequency (%)	
	Right	Left	Right	Left	Right	Left	Right	Left	Right	Left	Right	Left
At the 1^st ^posterior sacral foramina level	23	20	51.4	44.5	24	23	52.1	50	47	43	51.7	47.3
Below 1^st^ posterior sacral foramina	4	3	8.9	6.7	2	1	4.3	2.2	6	4	6.6	4.4
Above 1^st^ posterior sacral foramina	16	18	35.6	40	19	20	41.3	43.5	35	38	38.5	41.8
Between 1^st^ and 2^nd^ posterior sacral foramina	2	4	4.4	8.9	1	2	2.2	4.3	3	6	3.3	6.6

Type III sacrum was the most prevalent sacrum type according to the alpha angle of the SI joint on the sagittal axis, followed by Type II and Type I. Type III was observed more commonly in males than in females (Table 3, Figure 2). While the angle of the auricular surface was mostly above the first posterior sacral foramina in Type II, it was observed at the level of the first posterior sacral foramina in Type I and Type III (Table 2, Figure 2d).

**Table 2:** Classification of sacrum according to the alpha angle and the angle, level and surface area (cm2) measurement values of the auricular surface.

Classification of the Sacrum	Number (n)			Auricular surface angle (°)		Mean level of auricular surface angle	Surface area of auricular surface (cm^2^)	
	Total	Right	Left	Right	Left		Right	Left
Type I	9	4	5	161.92	165.73	1^st^ foramina level (7)	58.78	58.27
Type II	80	37	43	139.79	139.81	1^st^ foramina top (37)	52.42	47.50
Type III	93	50	43	119.60	116.16	1^st^ foramina level (91)	41.71	42.89

**Table 3:** Comparing the classification of sacrum according to the alpha angle by gender.

Classification of the Sacrum	Total			Male		Female		Total	
	Number (n)	Right	Left	Number (n)	%	Number (n)	%	Number (n)	%
Type I	9	4	5	4	4.4	5	5.4	9	4.95
Type II	80	37	43	29	32.2	51	55.4	80	44
Type III	93	50	43	57	43.8	36	39.1	93	51.1

In the typing made according to the alpha angle, the auricular surface area bilaterally, the length of the auricular surface, and the length of the right auricular surface angle did not differ significantly among sacrum types (p> 0.05) (Table 2, Figures 2a-2c).

The alpha angles on the right and left joint surfaces were greater in females than in males (p <0.05). No significant difference was observed between the sexes in terms of measurement values of the right and left surface area of the auricular surface, the length of the auricular surface, and the length of the auricular surface angle (Table 4, Figure 2).

**Table 4:** Comparison of the alpha angle on the joint surface, the length of the alpha angle, the length of the auricular surface and the auricular surface area by sex.

Parameters	Male (n=45)	Female (n=46)	P
Right alpha angle (°)	125.96 ± 15.17	133.28 ± 12.79	0.003
Left alpha angle(°)	126.67 ± 17.31	133.38 ± 15.31	0.045
Surface area of right auricular surface area (cm^2^)	47.3 ± 34.05	46.34 ± 40.11	0.727
Surface area of left auricular surface (cm^2^)	47.85 ± 34.94	44.02 ± 38.11	0.391
Right auricular surface length (cm)	4.9 ± .82	5.05 ± .83	0.385
Left auricular surface length (cm)	5.03 ± .82	4.85 ± .83	0.277
The length of the upper limit of the right auricular surface to the alpha angle (cm)	2 ± .43	2.63 ± 3.49	0.181
The length of the upper limit of the left auricular surface to the alpha angle (cm)	2 ± .56	1.91 ± .45	0.587

The mean value of the auricular surface length was greater in Group II than in Group III and Group I. The mean value of sacral width was 109.18 mm in the normal group (Group I), 111.14 mm in Group II, and 109.45 mm in Group III. The body width of the S1 vertebral body was greater in Group III compared to other groups, and Groups I and II had equal values (Table 5). In all groups, the auricular surface involved approximately 2.5 sacrums (Figure 3, Table 5).

**Table 5:** Grouping of sacrum according to the position of auricular surface in the sacrum and comparison of length measurements among the groups in mm.

Parameters	Group I. n=51	Group II. n=26	Group III. n=14
	Mean ± SD	Mean ± SD	Mean ± SD
Body width of the S1 vertebra corpus	52.05 ± 6.97	52.05 ± 6.28	54.35 ± 7.20
Auricular surface length	4.45 ± .86	5.01 ± 0.79	4.44 ± 0.44
Right auricular surface length	5.01 ± .91	4.98 ± 0.71	4.35 ± 0.42
Left auricular surface length	4.89 ± .81	5.04 ± 0.87	4.52 ± 0.46
Sacral width	109.18 ± 5.62	111.14 ± 4.59	109.45 ± 8.96

Group III (among males) and Group I (among females) showed greater values for sacral width, body width of the S1 vertebral body, and sacral length compared to other groups. There was no significant difference between the sexes in terms of these parameters (p> 0.05) (Table 6).

**Table 6:** Comparison of the sacral length, sacral width, and body width of the S1 vertebral body between the sexes in the grouping made according to the location of the auricular surface in the sacral vertebrae.

Sacrum Groups	Parameters	Male (mm) (Mean ± SD)	Female (mm) (Mean ± SD)	P
Group I n=51	Sacral width	108.86 ± 8.14	110.11 ± 9.95	0.756
	Body width of the S1 vertebral body	53.06 ± 6.86	55.81 ± 7.43	0.144
	Sacral length	103.52 ± 11.48	109.51 ± 10.67	0.093
Group II N=26	Sacral width	110.21 ± 4.56	112.07 ± 4.6	0.448
	Body width of the S1 vertebral body	50.94 ± 5.14	53.16 ± 7.28	0.362
	Sacral length	100.98 ± 11.82	101.49 ± 13.87	0.801
Group III n=14	Sacral width	109.33 ± 6.81	109.1 ± 5.3	0.797
	Body width of the S1 vertebral bodys	53.39 ± 7.23	51.31 ± 7.15	0.518
	Sacral length	115.27 ± 10.73	104.28 ± 10.3	0.083

In the present study, a statistically significant difference was found between right and left in the depth values of the impressions in the dorsal surface of the sacrum (p <0.05). The mean depth values of the right and left cranial impressions were greater than the mean depth values of the middle and caudal impressions. The right caudal impression depth was measured for 64 sacrums, and the left caudal impression depth was measured for 68 sacrums. The caudal impression had the smallest sacral impression measurements, and it was generally blunted or absent. Also, when the mean right and left anterior-posterior length values of the impressions in the dorsal surface of the sacrum were compared, the mean value of the cranial anterior-posterior length was greater than the mean value of the middle and caudal anterior-posterior length. The right caudal anterior-posterior length value was measured for 64 sacrums, and the left caudal anterior-posterior length value was measured for 67 sacrums. The caudal impression was generally blunted or absent and showed the smallest anterior-posterior length value (Table 7, Figure 4).

**Table 7:** Depth and anteroposterior length measurement values (mm) of impressions in the dorsal surface of the sacrum.

Parameters	Number (n)	Min.	Max.	Mean ± SD	P
Right cranial impression depth	91	1.16	26.07	10.89 ± 4.70	0.001
Right middle impression depth	91	.12	17.64	7.05 ± 3.55	0.001
Right caudal impression depth	64	.63	10.30	3.79 ± 2.02	0.001
Left cranial impression depth	91	1.51	19.48	10.06 ± 4.01	0.001
Left middle impression depth	90	.90	18.20	6.31 ± 3.47	0.001
Left caudal impression depth	68	.79	11.74	3.75 ± 2.26	0.001
Right cranial anterior-posterior length	90	5.70	23.97	13.59 ± 3.21	0.001
Right middle-posterior length	90	4.04	26.15	12.19 ± 4.23	0.001
Rigth caudal anterior-posterior length	64	1.61	12.25	7.84 ± 2.39	0.001
Left cranial anterior-posterior length	90	6.65	24.29	13.65 ± 3.48	0.001
Left middle-posterior length	89	3.54	22.69	12.08 ± 4.04	0.001

## Discussion

The sacrum carries weight and turns into a compact triangular bone. The sacrum receives the weight from the vertebral column located above it through the upper surface of the vertebral body of the S1 segment, as well as the joint facets that form the lumbosacral zygapophyseal joints. The magnitude of the forces received is proportional to the joint surface areas and also depends on the sacral joint angle. The load is transferred bilaterally to the ilium via the surface of the auricular surface over the sacroiliac joint [10,17,18]. The fusion of sacral segments imparts stability as well as efficiency in load transmission in a vertically oriented vertebral column [10]. It has been reported that SI joint pain is among the most common causes of low back pain, accounting for 15% to 25% of cases of axial low back pain [13,19,20]. The classification of sacrum allows for a biomechanically and clinically meaningful examination of the sacral structure. It also allows clinical correlation in patients with low back pain with the abnormal sacrum. The classification may also be used to provide additional information on the segments of the sacrum in situations where altered orientation and position of the auricular surface of the bone are associated with SI joint dysfunction [21-23]. What studies have yet to explore is if the basic anatomic size and shape of the SI joint may play a role in the development of SI joint pain and dysfunction [24]. In the current study, special care was exercised to classify and further explain interpersonal variability in SI joint morphology.

Mahato grouped the sacrum according to the position of the auricular surfaces. In Group I (normal sacra), the load was primarily routed through the first 2.5 segments at the auricular surfaces that are located exactly at the level of the S1, S2, and upper S3 segments. These sacra exhibited attenuation of their sacral segment sizes below with a quick narrowing of the lateral borders. The Group II sacra demonstrated stable upper segments (S-1 and S-2) with strong features of load transmission at their costal and transverse elements and sacral bodies. Weight transfer in these sacra occurs chiefly at the upper vertebral levels due to the superior position of the auricular surfaces. This weight is transferred laterally to the two sacroiliac joints. The Group III (low-down) auricular surfaces displayed increased stability and strength at the lower (S-2 and S-3) sacral segments. The occasional presence of exaggerated gaps between the S1 and S2 segments in the low-down sacra indicates that more weight is transmitted through the lower segments of these vertebrae, which obviates the need for complete fusion of the S1 segment. This can be viewed as a condition of incomplete incorporation of the S1 segment in the sacral “stockpiling” due to already stable and efficient lower segments. Mahato found that the average length of the auricular surfaces in Group II was smaller compared to Group I and Group III. The crucial position of the auricular surfaces determines the load-bearing pattern in the sacrum, including the specific sacral segments that are involved in the transmission of load toward the hip bone [10].

Researchers have stated that some anatomical parameters related to the sacrum, especially S1, are very important for the spinal instrumentation procedure of spine surgeons [25,26]. The investigators reported that the mean value of the transverse diameter of the S1 vertebral body was 52.6 ± 0.70 mm [25], 51.1 mm [27], 49.4 mm [26], and 49.33 ± 6.74 mm [28]. In his study, Mahato found that the mean S1 vertebral body transverse diameter was quite low in Group II and that the sacral width in this group was similar to that in Group I [10]. The mean S1 vertebral body transverse diameter was 52.81 mm in the current study. In addition, the mean S1 vertebral body transverse diameter was larger in males and females in Group III and Group I, respectively, than in the other groups.

Mahato stated that the position of the auricular surface in the sacrum differs among individuals, and the auricular surface in all groups covered approximately 2.5 sacrums [10]. Consistently, the auricular surface covered approximately 2.5 sacrums in all groups in the present study.

In their retrospective study, Jesse et al. classified variations in the joint morphology of the SI joint into three types according to the alpha angle on the sagittal axis of the joint in patients with sacroiliac joint pain. Based on their classification, type II was the most common sacrum type. They also found that type II was more common in males (74%) than in females (67%). In addition, they concluded that the variability in the synovial surface morphology of the SI joint can prepare the ground for the development of low back pain [13]. In the current study, type III was the most common sacrum type, followed by type II and type I. Type III was slightly more common in males than in females.

The morphology of the SI joint shows some variability in the development stages of the sacrum. The sacroiliac joint surfaces are smooth in the first years of life and lose their flat surface over time [6]. The impressions on the dorsal surface of the sacrum and Tuberositas ossis sacri on the articular surface of the ilium occur in response to stress and vary among individuals. At the same time, the impressions create a functional locking between the ilium and sacrum, and this locking serves to optimize joint stability by limiting movement occurring in the joint. Thus, dislocations that may occur in the joint are greatly reduced with increased stability of the SI joint [4,6]. Gray suggested that pain may result from minimal movements that cause collisions between the complementary sacral recess and impressions of the SI joint, accompanied by limitation of motion in the joint [29]. Postacchini et al. reported little interest in the extra-articular part, which they called the impressions on either side of the dorsum of the sacrum and the posteroinferior of the ilium, and the extra-articular components in contact with these impressions to form the extra-articular spaces where the posterior sacroiliac ligaments enter. They found that the depth values of cranial impressions in the dorsal surface of the sacrum were significantly higher than the depth values of the middle and caudal impressions. They also found a significant difference between the anterior-posterior length values of cranial and caudal impressions in the dorsal surface of the sacrum [2]. Interpretation of CT or MRI images is important in the anatomical evaluation of the sacroiliac region. They stated that the sacral and iliac components may have different morphometric properties on both sides of the bone in individuals and that the related structures adapt to each other to form the bone complex that enables the attachment of the ilium to the sacrum and the coupling of the auricular surfaces. It has been reported in the literature that the sacroiliac extra-articular part of the sacroiliac region has unique characteristics [30,31]. Prossopoulos et al., the variation they call "Iliosacral complex"; stated that in the lower posterior part of the S1 level, where the usually large cranial sacral impression is located, it may be due to the protrusion of the ilium in front of this impression. The variation they call "Semicircular defect" is emphasized that it is usually located at the S2 level and is probably due to the mild depression of the ilium in front of the medial sacral depression [30]. In the current study, a statistically significant difference was found when the mean depth values of the right cranial, middle, and caudal impressions and the mean anterior-posterior length values of the left cranial, middle, and caudal impressions were compared individually (p <0.05). The mean depth value of cranial impressions was greater than the mean depth values of middle and caudal impressions. In addition, the cranial anterior-posterior mean length was greater than the middle and caudal mean length values in our study. The results of Postacchini et al. support our findings.

Esenkaya et al. reported a mean sacral width of 110.3 mm [27]. Asher et al. reported the mean sacral width as 104 mm and 106.4 mm, respectively, in males and females and found that there was no statistically significant difference between sexes [32]. Başaloğlu et al. found the mean sacral width as 108.4 mm in females and 102.2 mm in males and reported a statistically significant difference between sexes (p = 0.001) [25]. Mishra et al. found that the sacral width in 116 dry os sacrum of the Agra region was 105.34 mm in males and 105.79 mm in females [33]. Koç et al. reported the mean sacral width as 111.67±6.57 mm [28]. Mahato observed that the mean sacral width of Group II was similar to that of Group I [10]. In this study, the mean sacral width (109.92 mm) was similar in all three groups. Davivongs reported that the sacral length in Australian aborigines was 96.52 mm in males and 88.12 mm in females [34]. Similarly, Mishra et al. found that the mean sacral length was greater in males (107.53 mm) than in females (90.58 mm). Also, they reported that sacral length measurements in male sacrums in the Agra region were greater than those in the Varanasi region, but the opposite was true in females [33].

Esenkaya et al. reported the mean sacral length as 105.7 mm [27]. Koç et al. found the mean sacral length as 93.49±13.55 mm [28]. Mahato reported mean sacral length values of 105.05 mm, 94.82 mm, and 108.40 mm, respectively, in Group I, Group II, and Group II [10]. In the current study, the mean sacral length was 108.21 mm in Group I, 101.24 mm in Group II, and 106.34 mm in Group III. Comparing the sacral length between the sexes, the mean sacral length was 103.52 mm in males and 109.51 mm in females from Group I. In Group II, the mean sacral length was 100.98 mm for males and 101.49 mm for females. In Group III, the mean sacral length was measured as 115.27 mm in males and 104.28 mm in females. Our findings are largely similar to those reported in the literature, and we believe that small differences in the numerical data may be due to the differences in the regions where the sacrums were obtained, the methods used for measurements as well as sex differences.

Koç et al. reported that the mean surface area of the auricular surface was 1028.15 ± 232.92 mm on the right side and 1042.45 ± 220.72 mm on the left [28]. Mahato stated that the auricular surface area defines the magnitude of the weight transmitted to the hip bone, and it may also be important in carrying the load on the sacrum. Mahato reported no significant difference between the groups in terms of auricular surface areas [10-12]. Similarly, in the present study, no significant difference was observed between the groups in terms of the auricular surface areas.

Limitations of the study and future directions

This study was performed on dry human adult sacral bones. Since the study material included dry sacra, we considered that there would be no need to make comparisons with radiological studies. It is important for surgeons using fluoroscopy to have a detailed knowledge of bone anatomy and to have a good knowledge of CT anatomy in order to preoperatively define the morphology of the sacroiliac region of their patients. In order to perform a successful operation, it is important to have a comprehensive knowledge of the bone anatomy and to have a perfect knowledge of the CT anatomy of the relevant part. We think that comparing the dry sacroiliac complex and radiological studies in future studies will contribute to surgery and anatomical literature.

## Conclusions

It could be of immense value for clinicians to assess and understand the gross morphology of sacra in light of the positions of their auricular surfaces. The grouping and classification system used in this study classifies variations associated with sacral anatomy along a common-to-rare anatomical spectrum that may provide relevant information needed in a clinical and biomechanical context. The findings of the current study may provide additional specific information about variations in the sacral anatomy for use for anthropological or forensic purposes.

Main points

The sacral index was established as a definitive cut-off value for sex determination. The sacral index values were 90.5 to 106 mm in males and 104.8 to 131 mm in females. Although the auricular surface covered approximately 2.5 sacrums in all groups, there was a difference in the position of the auricular surface in the sacral vertebrae. The surface area of the facies auricularis was larger in males than in females. It was determined that the impressions on the dorsal surface of the sacrum differed on the right and left sides of the individuals. Evaluation of the detailed morphology of the sacral bone showed a highly variable morphology among individuals.
